# Animal models of mechanisms of SARS‐CoV‐2 infection and COVID‐19 pathology

**DOI:** 10.1111/bph.15143

**Published:** 2020-07-19

**Authors:** Simon J. Cleary, Simon C. Pitchford, Richard T. Amison, Robert Carrington, C. Lorena Robaina Cabrera, Mélia Magnen, Mark R. Looney, Elaine Gray, Clive P. Page

**Affiliations:** ^1^ Department of Medicine UCSF San Francisco CA USA; ^2^ Sackler Institute of Pulmonary Pharmacology, Institute of Pharmaceutical Science, School of Cancer and Pharmaceutical Sciences King's College London London UK; ^3^ Covance Laboratories Limited Huntingdon UK; ^4^ National Institute for Biological Standards and Control Herts UK

## Abstract

The coronavirus disease 2019 (COVID‐19) pandemic caused by SARS‐CoV‐2 infections has led to a substantial unmet need for treatments, many of which will require testing in appropriate animal models of this disease. Vaccine trials are already underway, but there remains an urgent need to find other therapeutic approaches to either target SARS‐CoV‐2 or the complications arising from viral infection, particularly the dysregulated immune response and systemic complications which have been associated with progression to severe COVID‐19. At the time of writing, in vivo studies of SARS‐CoV‐2 infection have been described using macaques, cats, ferrets, hamsters, and transgenic mice expressing human angiotensin I converting enzyme 2 (ACE2). These infection models have already been useful for studies of transmission and immunity, but to date only partly model the mechanisms involved in human severe COVID‐19. There is therefore an urgent need for development of animal models for improved evaluation of efficacy of drugs identified as having potential in the treatment of severe COVID‐19. These models need to reproduce the key mechanisms of COVID‐19 severe acute respiratory distress syndrome and the immunopathology and systemic sequelae associated with this disease. Here, we review the current models of SARS‐CoV‐2 infection and COVID‐19‐related disease mechanisms and suggest ways in which animal models can be adapted to increase their usefulness in research into COVID‐19 pathogenesis and for assessing potential treatments.

**Linked Articles:**

This article is part of a themed issue on The Pharmacology of COVID‐19. To view the other articles in this section visit http://onlinelibrary.wiley.com/doi/10.1111/bph.v177.21/issuetoc

**Abbreviations**
ARDS: acute respiratory distress syndrome
DIC: disseminated intravascular coagulation
NETs: neutrophil extracellular traps
SARS: severe acute respiratory syndrome

## 1 INTRODUCTION

Animal models are necessary in the development of all drugs and therapeutic agents to demonstrate efficacy and safety, as well as providing essential information on route of administration, pharmacokinetics, and pharmacodynamics and to identify key mechanisms driving pathology in vivo. The pandemic of coronavirus disease 2019 (COVID‐19), the disease manifestation of SARS‐CoV‐2 infection, has led to the rapid development of animal models of SARS‐CoV‐2 infection which have already provided insights into the natural history of the disease and, together with data from previous studies, have permitted identification of potential antiviral approaches identified in studies of other viral infections (Alexander et al., 2020).

Notably, at the time of writing (late April 2020), studies using animal models have already provided some evidence that immunity might confer protection from reinfection (Bao et al., 2020) and that convalescent plasma might decrease viral burden (Chan et al., 2020). Animal studies have also confirmed the susceptibility of domestic cats to infection (Shi et al., 2020) and have provided evidence of the importance of ACE2 in enabling infection (Bao et al., 2020).

**FIGURE 1:**
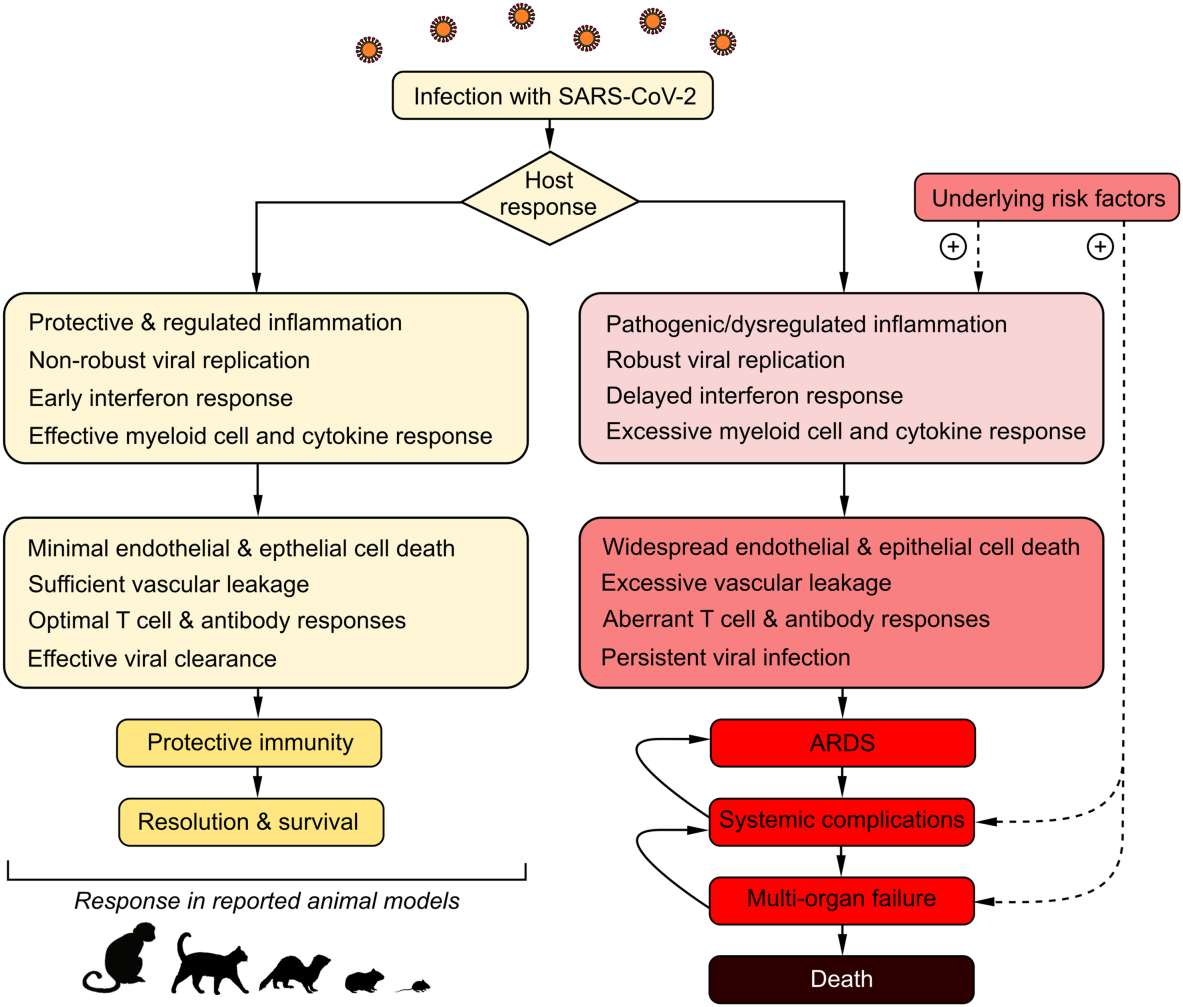
Model of factors driving progression to mild or severe COVID‐19. Flow diagram representing a model of protective versus dysregulated responses to SARS‐CoV‐2 infection. Most reported animal models of SARS‐CoV‐2 infection are likely to involve protective immunity and resolving pathology. Risk factors and mechanisms implicated in driving severe responses to SARS‐CoV‐2 infection provide insights into how to push models towards replicating pathological responses. Adapted from Channappanavar and Perlman (2017)

**FIGURE 2:**
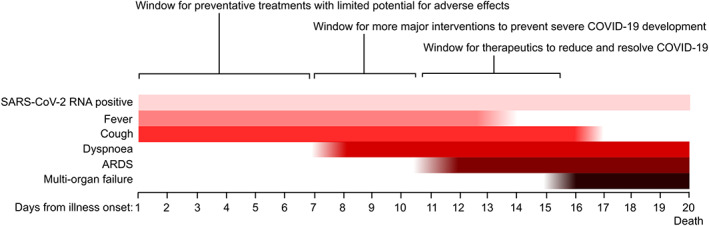
Windows for clinically feasible application of different types of therapeutic agents for COVID‐19. Time course of symptomatic progression in lethal COVID‐19 simplified from Zhou et al. (2020), annotated with time windows indicating when different therapeutic interventions that might realistically be applied

These animal models are well suited for proof of concept studies into the efficacy of potential vaccines or antivirals. However, each model system has its drawbacks, and at the time of writing (April 2020), no reported animal model of SARS‐CoV‐2 infection fully reproduces every key feature of severe COVID‐19 (Table 1 and Figure 1). This deficit in preclinical modelling is important as, until vaccines are widely available, there is a particularly urgent need to identify potential treatments for patients who already have established SARS‐CoV‐2 infection and who are at risk of progressing to severe COVID‐19 requiring hospitalisation and the need for respiratory support in a high dependency or intensive care unit. The ideal evidence from preclinical efficacy studies of potential therapeutic interventions to prevent or promote resolution of severe COVID‐19 in patients who are already symptomatic will be meaningful improvements in clinically relevant endpoints in models which deterministically progress to severe disease following infection with SARS‐CoV‐2. Interventions should also be applied at realistic time points in these models (Figure 2). This ideal standard of evidence will of course have to be balanced with practical and ethical considerations (Table 2).

**TABLE 1:** Summary of reported animal models of SARS‐CoV‐2 infection

	Model organism									
	Cynomolgus macaque	Rhesus macaque			Cat	Ferret			Golden Syrian hamster	hACE2 mouse
Reference	(Rockx et al., 2020)	(Bao, Deng, Huang, et al., 2020)	(Munster et al., 2020)	(Yu et al., 2020)	(Shi et al., 2020)	(Shi et al., 2020)	(Kim et al., 2020)	(Blanco‐Melo et al., 2020)	(Chan et al., 2020)	(Bao, Deng, Huang, et al., 2020)
Inoculation	10^6^ TCID_50_ (i.n. + i.t.)	10^6^ TCID_50_ (i.t.)	2.4 × 10^6^ TCID_50_ (i.n. + i.t. + p.o. + o.u.)	10^6^ TCID_50_ (i.t.)	10^5^ PFU (i.n.)	10^5^ PFU (i.n.)	10^5.5^ TCID_50_ (i.n.)	10^5^ PFU (i.n.)	10^5^ PFU (i.n.)	10^5^ TCID_50_ (i.n.)
Lung inflammation	Yes (histology and superficial)	Yes, limited (histology)	Yes (histology and superficial)	Yes (histology)	Yes (histology)	Yes, severe (histology)	Yes, limited (histology)	None reported	Yes (histology)	Yes, limited (histology and superficial)
Alveolar/capillary barrier dysfunction	Alveolar flooding (histology, only in young animals)	Interstitial pneumonia (radiology and histology)	Infiltrates (radiology) oedema (lung weight as % of body weight) alveolar flooding, hyaline membranes (histology)	Interstitial pneumonia and alveolar flooding (radiology and histology)	Alveolar flooding (histology)	None reported	None reported	None reported	Severe alveolar flooding and lung consolidation resolving by 14 d.p.i. (histology)	None reported
Physiological gas exchange impairment	None reported	None reported	Increased respiratory rate	None reported	None reported	None reported	None reported	None reported	Increased respiratory rate	None reported
Systemic Inflammation and complications	None reported	None reported	Neutropenia, anaemia (CBC), not detected (serum cytokine analysis)	Decreased lymphocytes (CBC) asthenia	None reported	None reported	Elevations in body temperature	None reported (upper resp. tract IL‐6, IL1RA persistently up‐regulated)	Weight loss, resolving inflammation (lung chemokine and cytokine analysis)	Temporary body weight loss
Mortality	None reported	None reported	None reported	None reported	1 juvenile cat died at 3 d.p.i	None reported	None reported	None reported	None reported	None reported

Abbreviations: d.p.i., days post infection; i.n., intranasal; i.t., intratracheal; o.u., oculus uterque (applied to both eyes); p.o., per os (by mouth); PFU, plaque forming units; TCID_50_, median tissue culture infective dose.

**TABLE 2:** Major advantages and disadvantages of different animal models of SARS‐CoV‐2 infection

Animal model	Advantages	Disadvantages
Macaque	• Phylogenetically close to humans • Used in viral infection research	• Low throughput • More advanced cognition presents additional ethical issues
Cat	• Lethality with pulmonary oedema reported • Natural infections reported	• Not widely used in pathology studies • Aggression and unpopularity of use as laboratory animals
Ferret	• Permit study of cough and fever symptoms • Used in viral infection research	• Unclear whether serious lung infection and oedema can be caused by SARS‐CoV‐2
Hamster	• High homology with human in terms of ACE2 • Demonstrate substantial lung inflammation and injury	• Not widely used • Limited research tools available
Mouse	• Wide range of research tools available • Immune responses highly characterised • Higher throughput	• Transgenic expression of hACE2 or viral adaptation required • Some major differences in lung and immune physiology compared to humans

SARS‐CoV‐2 has zoonotic origins (Andersen, Rambaut, Lipkin, Holmes, & Garry, 2020), but several major factors complicate the study of this virus in model organisms. These include lack of infectivity of clinical isolates of SARS‐CoV‐2 in some model species including mice and dogs (Bao, Deng, Huang, et al., 2020; Shi et al., 2020) and an absence of the persistent infection, immunopathology, severe acute respiratory distress syndrome, and systemic complications which characterise COVID‐19 clinically. Furthermore, SARS‐CoV‐2 predominately causes severe COVID‐19 in older people with co‐morbidities (Wang et al., 2020), presenting a construct validity problem with attempted use of young and immunologically naïve laboratory animals in COVID‐19 testing.

Scientists developing animal models therefore face a challenging set of trade‐offs as well as a sense of urgency. Despite the pressing needs for treatments, design and implementation of preclinical studies for COVID‐19 should maintain quality to produce meaningful results, avoid needless duplication, and avoid undue reduction in consideration of animal welfare issues (London & Kimmelman, 2020). Here, we review the mechanisms involved in COVID‐19 development and reported approaches to model SARS‐CoV‐2 infection responses. We then identify key areas in which studies using animal models might improve the reproduction of important characteristics of human COVID‐19 to better help in the identification and assessment of new therapeutic interventions.

## 2 BACKGROUND ON SARS‐COV‐2 AND COVID‐19

New modelling approaches are needed to improve our understanding of SARS‐CoV‐2 and COVID‐19 because the virus has only recently infected humans and because aspects of the disease are different to previously described syndromes developing as a result of viral infections including SARS. Fortunately, clinical and preclinical data related to the COVID‐19 pandemic have been disseminated at a never‐before‐seen rate.

SARS‐CoV‐2 is a positive‐sense single‐stranded RNA coronavirus thought to be the descendant from a bat coronavirus which spilled over to infecting humans after infecting an intermediate host, potentially a pangolin (Andersen et al., 2020; Zhang, Wu, & Zhang, 2020). The genome of SARS‐CoV‐2 is 79% homologous with that of SARS‐CoV which caused severe acute respiratory syndrome (SARS) outbreaks in 2003 (Lu et al., 2020). Studies indicate that, as with SARS‐CoV, the Spike glycoprotein expressed by SARS‐CoV‐2 undergoes activating cleavage by host proteases on epithelial surfaces which permits high affinity interaction of Spike with host epithelial ACE2 (Bao, Deng, Huang, et al., 2020; Hoffmann et al., 2020). When it is bound by Spike, cleavage of ACE2 by further host proteases such as TMPRSS2 allows viral entry into host epithelial cells for viral replication (Hoffmann et al., 2020). Damas et al. (2020) assessed ACE2 polymorphism and similarity to human ACE2 in 410 vertebrates, including 252 mammals. Based on conservation of 25 amino acids that are essential for interaction between ACE2 and SARS‐CoV‐2, a scoring system that predicts the likelihood of infectivity was devised. Although experimental data would be required to substantiate the merit of this system, this study does provide a rationale for choice of animal species to study the infectivity, pathogenesis, and treatment of COVID‐19 and suggested that species such as Old World monkeys would be highly susceptible to infection, while most rodents are less likely to be infected.

SARS‐CoV‐2 and SARS‐CoV exhibit similar stability outside of the body (van Doremalen et al., 2020) but differ in their incubation and transmission kinetics. Compared with SARS‐CoV, SARS‐CoV‐2 appears to replicate more rapidly in the upper respiratory tract (Zou et al., 2020), where it achieves transmission in asymptomatic carriers (Bai et al., 2020), a factor which is likely to contribute to the rapid global spread of COVID‐19.

The reported clinical symptoms of COVID‐19 range from mild to critical (Wang et al., 2020). Mild disease varies from no symptoms to mild pneumonia. Severe disease is characterised by moderate to severe pneumonia. Critical COVID‐19 involves diagnosis of acute respiratory distress syndrome (ARDS), septic shock, and/or multi‐organ failure (Wu & McGoogan, 2020). Risk factors including advanced age, male sex, obesity, diabetes, and immunodeficiency predispose towards development of severe or critical COVID‐19. SARS‐CoV‐2 appears to be less likely to cause severe disease than SARS‐CoV (Ruan, 2020), a factor which is likely also to allow a greater spread of SARS‐CoV‐2 and may also present an additional challenge for development of animal models.

Pathology of COVID‐19 is centred around immunopathology with persistent lung infection leading to ARDS, and clinical progression follows a time course characteristic of a dysregulated viral immune response (Gattinoni, Coppola, Cressoni, Busana, & Chiumello, 2020; Tian et al., 2020; Xu et al., 2020) (Figure 1). It is clear, however, that critical cases of COVID‐19 involve damage to other systems in the body which may both be a result of ARDS and may also be causative of greater lung injury. Notably, COVID‐19 has been associated with a coagulopathy driven by inflammation and characterised by elevated fibrinogen and D‐dimer levels indicating increased thrombin generation and fibrinolysis (Tang et al., 2020; Zhang et al., 2020). Patients suffer from increased thrombotic risk against which standard prophylactic anticoagulants appear only partly effective (Llitjos et al., 2020), and there is evidence that platelets, autoantibodies and neutrophil extracellular traps (NETs) may be involved in the pathogenesis (Barnes et al., 2020; Bikdeli et al., 2020; Zhang et al., 2020; Zuo et al., 2020).

The ARDS presentation within COVID‐19 is heterogenous and may involve both gas exchange and perfusion abnormalities (pulmonary dead space) to extents that differ between patients (Gattinoni et al., 2020). Additionally, hyperactivation of inflammatory responses can result in a cytokine storm which may kill via exacerbation of multi‐organ failure and lung inflammation or myositis (Blanco‐Melo et al., 2020; Sarzi‐Puttini et al., 2020). Reproduction of these key processes driving severe pathology and mortality in COVID‐19 will validate animal models for severe COVID‐19 research.

## 3 ANIMAL MODELS OF SARS‐COV‐2 INFECTION

### 3.1 Non‐human primates

Non‐human primates are close in phylogeny to humans which makes them particularly important for use in vaccine development. At the time of writing, non‐human primate models of SARS‐CoV‐2 infection have been reported using rhesus macaques (Bao, Deng, Gao, et al., 2020; Munster et al., 2020; Yu et al., 2020) and cynomolgus macaques (Rockx et al., 2020). For studies of SARS‐CoV, African green monkeys, common marmosets, squirrel monkeys, and moustached tamarins have also been used (Gong & Bao, 2018). Although non‐human primate models can resemble human systems more closely than models which use more phylogenetically distant animals, it is important to note that these studies frequently use limited numbers of animals in their experiments (as low as one or two animals per group), and so their results should be interpreted with due caution (Curtis et al., 2018). Investigators using these intelligent animals do however have limited numbers of non‐human primates available for terminal proof of concept pathology studies, especially as many of these animals will soon be needed for vaccine tests, and so experimental designers are forced to make trade‐offs between group sizes in early pathology studies and those in later preclinical trials of therapeutics.

The most convincing demonstration of an animal model of COVID‐19 has been reported by Munster et al. (2020). Rhesus macaques were inoculated intranasally, intratracheally, by mouth, and onto both eyes. Pulmonary infiltrates were observed radiologically, and oedema was measured gravimetrically, although superficial inspection found lesions to be focal and sporadic. Alveolar flooding with the presence of hyaline membranes, which are rarely seen in other animal models of ARDS (Matute‐Bello et al., 2011), were observed in lung histology. This was accompanied by an irregular breathing pattern and increased respiratory rate in some animals suggestive of hypoxaemia, although this readout may also be a marker of pain or distress. Together these results suggest some degree of ARDS development. However, gas exchange impairment was not measured, and serum cytokine analysis detected no consistent evidence of systemic inflammation.

As age is a major risk factor for severe COVID‐19, the response of aged rhesus macaques (~15 years old) to intratracheal inoculation with SARS‐CoV‐2 has also been compared with that of younger controls (3–5 years old) (Yu et al., 2020). This report suggests that there are age‐related increases in viral load 7 days after inoculation. Radiology and histology were indicative of mild interstitial infiltrates in younger animals with signs of more severe oedema including alveolar flooding in aged macaques. Although preliminary and largely qualitative, this study suggests that aged macaque models may be useful for modelling more severe disease.

Rhesus macaques have also been used to test whether seroconversion provides protective immunity against SARS‐CoV‐2. In one reported study, two animals were inoculated intratracheally with SARS‐CoV‐2 and then challenged again 28 days later (Bao, Deng, Gao, et al., 2020). Lack of viral shedding after re‐challenge in both macaques suggested development of protective immunity. This study should not be overinterpreted, however, as other studies suggests low, or even undetectable, titres of neutralising antibodies in previously infected patients (Wu et al., 2020), and the Korean Centre for Disease Control has reported reinfection of patients with previous COVID‐19. Limited signs of lung inflammation and pneumonia were also demonstrated in this macaque study.

Rockx et al. (2020) challenged both young adult (4–5 years) and aged (15–20 years) cynomolgus macaques with SARS‐CoV‐2 using a combined intranasal and intratracheal inoculation approach. Two animals in each age group were autopsied at 4 days post infection, and limited focal lesions were observed in the lungs of a young adult and an aged macaque. The lesions in the lungs of the young adult showed alveolar flooding and hyaline membrane formation with other signs of diffuse alveolar damage which co‐localised with SARS‐CoV‐2 nucleocapsid staining. These observations are promising signs that a modified approach in cynomolgus macaques where viral infection affects a greater proportion of the lungs may be useful in modelling mechanisms driving severe COVID‐19.

### 3.2 Ferrets

Ferrets are useful in both studies of viral transmission and the pharmacology of the most frequently reported symptom of SARS‐CoV‐2 infection as, unlike mice and rats, they exhibit a cough reflex. There are also ferret models used to study cystic fibrosis (Sun et al., 2010). Ferret studies also have potential veterinary and zoonotic relevance as an outbreak of SARS‐CoV‐2 infections with respiratory symptoms has been reported in minks, related to ferrets within the Mustilidae family, in two farms in the Netherlands (Dutch Parliament report, 2020).

Following inoculation with SARS‐CoV‐2, ferrets have been shown to develop symptoms similar to those described in human COVID‐19, namely, elevated body temperature suggestive of pyresis, reduced activity and appetite, and coughing between 2 and 12 days post infection (Kim et al., 2020; Shi et al., 2020). Histologically, SARS‐CoV‐2 infected ferret lungs have exhibited severe pulmonary lymphoplasmacytic perivasculitis and vasculitis at 13 days post infection (Shi et al., 2020).

Transmission of SARS‐CoV‐2 was studied by placing naïve ferrets in direct or indirect contact with inoculated ferrets (Kim et al., 2020). It was observed that all naïve ferrets placed in direct contact with infected ferrets displayed symptoms of infections (elevations in body temperature and reduced activity) 2–6 days post infection. However, ferrets placed in indirect contact with inoculated animals through separation with a partition that allowed air to move between enclosures did not show any symptoms, although some tested positive for viral RNA indicative of airborne transmission. Lung histology of inoculated ferrets in this study only showed mild signs of inflammation at 4 days post infection.

Ferrets have also been used for longitudinal studies of immune responses to SARS‐CoV‐2 infection by intranasal inoculation with SARS‐CoV‐2 and repeated measurements of upper respiratory tract gene transcripts from nasal washes (Blanco‐Melo et al., 2020). These studies showed a lower magnitude of upper airway immune responses relative to influenza A infection and the induction of a unique SARS‐CoV‐2 gene signature ontologically enriched for cell death and leukocyte activation‐associated transcripts. Tracheal cell analysis also showed that SARS‐CoV‐2‐unique transcripts were also associated with haematopoietic progenitors, suggestive of extramedullary haematopoiesis at the infection site. However, importantly, this study did not report any lower respiratory or systemic pathological findings, and the lack of demonstrated pulmonary replication and oedema in ferret SARS‐CoV‐2 infection models suggests a major limitation of ferrets in the study of lung pathology.

### 3.3 Cats

Although cats are not widely used to study respiratory diseases, the close association of humans and domestic cats means that investigation into SARS‐CoV‐2 infection and transmission in cats is important, although the relationship of domestic cats with humans in turn complicates their use as laboratory animals. It is notable here that domestic cats, as well as zoo‐housed tigers which are also in the Felidae family, have reportedly tested positive in the United States suggesting veterinary and zoonotic importance of feline SARS‐CoV‐2 studies (Center for Disease Control Report, 2020).

To study transmission of SARS‐CoV‐2 two pairs (one inoculated and one naïve in each pair) of sub‐adult (aged 6–9 months) and three pairs of juvenile cats (aged 10–14 weeks) were housed together, with faecal samples analysed for the presence of viral RNA to confirm successful infection. In both the sub‐adult and juvenile cats, only one out of the three naïve cats had viral RNA detected in the faecal samples, suggestive of some transmission, albeit more limited than in ferrets. It is notable here that the researchers were unable to perform nasal washes on the sub‐adult cats due to the aggression of the cats.

Also notable was the reported death of one of the inoculated juvenile cats at 3 days post infection during this study, suggestive of the provocation of severe disease in a young animal. Lung histology on autopsy showed pronounced alveolar flooding, suggesting pulmonary oedema development. Further study of whether severe disease can be reproduced in cats may be useful for severe COVID‐19 efficacy testing or for veterinary medicine development.

### 3.4 Hamsters

Hamsters have been used for a range of medical research studies, and importantly show a relatively high degree of homology with humans within the region of ACE2, which is involved in interaction with the ACE2‐binding domain of SARS‐CoV‐2 Spike (Chan et al., 2020).

Chan et al. (2020) inoculated Golden Syrian hamsters intranasally with SARS‐CoV‐2. Viral replication occurred in the lungs of infected hamsters, and lungs also developed marked lesions of pulmonary oedema, inflammation, and cell death as assessed histologically. Inoculated hamsters lost weight, showed an increased respiratory rate, and could infect co‐housed hamsters suggesting utility as a model for studying transmission. The infected co‐housed hamsters showed similar signs of lung pathology but did not lose weight, suggesting that inoculated hamsters had greater severity due to a higher amount of virus delivered to lungs.

Inflammation was also measured in this study using quantitative PCR on lung samples, demonstrating an early response of IFN and an elevation of IL‐6. Together these data suggest that hamsters might be highly useful in modelling mechanisms of COVID‐19. However, inflammation and lung pathology resolved by 14 days post infection, suggesting that the hamster responses were reflective of a resolving inflammation and successful host defence as opposed to the dysregulated responses that are associated with severe COVID‐19.

### 3.5 Mice

Mice are widely used in studies of immunology and lung injury and have a highly characterised immune system, rapid breeding cycle, and can be used with a range of readily available research tools. Importantly, the size and rapid breeding and growth of mice is useful for accelerating the completion of studies with group numbers high enough for statistical testing of efficacy of potential interventions.

However, the Spike proteins of SARS‐CoV‐2, and of its relative, SARS‐CoV, are thought to have insufficient affinity for the murine ACE2 entry receptor for infection of mice (Wan, Shang, Graham, Baric, & Li, 2020). Clinical isolates of SARS‐CoV have therefore been adapted by serial passage in the respiratory tract of mice to produce related viruses (MA15 and v2163) that can cause lung injury and mortality in wild‐type mice (Day et al., 2009; Roberts et al., 2007), with pathological exacerbation reported in aged or immunodeficient mice (Graham et al., 2012).

At the time of writing, there are no reports of mouse‐adapted SARS‐CoV‐2, and it is not known whether naturally occurring mutations in the SARS‐CoV‐2 Spike ACE2‐binding domain found in clinical isolates have altered pathogenicity in mice (Ou et al., 2020). Mouse adaptation of SARS‐CoV‐2 would be useful for the acceleration of mouse testing but may prove difficult as SARS‐CoV‐2 Spike appears to have evolved high affinity for human ACE2 at the expense of lower affinity for ACE2 in other organisms (Wan et al., 2020).

Fortunately, the issue of low Spike‐murine ACE2 affinity has been addressed in previous SARS‐CoV studies leading to the development of the K18‐hACE2 mouse, in which transgenic human ACE2 (hACE2) expression is driven in mouse epithelial cells under the control of the human cytokeratin 18 (K18) promoter (McCray et al., 2007). K18‐hACE2 mice were treated with doses of SARS‐CoV (2.3 × 10^4^ PFU) which did not induce pathology in mice without transgene expression. The transgenic mice replicated virus in their lungs, experienced weight loss, and developed severe histological evidence of lung inflammation and mortality at around 4 days post infection (McCray et al., 2007).

Initial studies preprints show that SARS‐CoV‐2 can also infect another strain of mice expressing hACE2 driven by the mouse *Ace2* promoter in a transgene‐dependent manner (Bao, Deng, Huang, et al., 2020; Yang, Deng, et al. 2007). In these mice, inoculation with SARS‐CoV‐2 at 10^5^ TCID_50_ caused weight loss, antibody responses, and both superficial and histological evidence of lung inflammation in a hACE2 transgene‐dependent manner, although lung injury was limited and not quantified (Bao, Deng, Huang, et al., 2020). In contrast to previous SARS‐CoV experiments, however, no mortality was reported with SARS‐CoV‐2 infection in hACE2 mice, perhaps related to the lower virulence of SARS‐CoV‐2 observed in humans, although only one dose of virus was studied. Further adaptations of the experimental approach to infecting transgenic mice may be required to study lethal lung injury resulting from SARS‐CoV‐2 infection.

Another issue for further studies is whether the tissue distribution and surface expression levels of hACE2 in hACE2 mice fully replicate those in humans, as murine ACE2 expression appears to be highly localised to bronchial epithelium in mice (Sodhi et al., 2019; Sun, Gu, Ma, & Duan, 2020), with ACE2 perhaps more generally distributed in human lungs (Hamming et al., 2004) and when transgenic hACE2 expression is driven by the mouse *ACE2* promoter in mouse lungs (Bao, Deng, Huang, et al., 2020). There are also species differences in distribution of ACE2 expression outside the lungs which may have implications for systemic responses to SARS‐CoV‐2 infection. Additionally, ACE2 down‐regulation may play a role in disease progression (Kuba et al., 2005), and hACE2 have both human and non‐Spike reactive murine ACE2 and so may be resistant to ACE2 down‐regulation related complications. The targeted insertion of human ACE2 into the endogenous mouse locus may therefore be useful in generating a better model of severe COVID‐19.

### 3.6 Other species

SARS‐CoV‐2 probably originated from a coronavirus infecting bats (Andersen et al., 2020; Zhang, Wu, & Zhang, 2020). Although bats are not commonly used as model organisms they are of particular research interest because (a) they appear to be the originating species of many particularly deadly zoonotic viruses, and (b) their immune systems have evolved to tolerate persistent infections with viruses with higher virulence in other species, which possibly accelerates viral evolution (Brook et al., 2020; Rabi, Al Zoubi, Kasasbeh, Salameh, & Al‐Nasser, 2020).

These features of bats mean that live wild bats should probably be avoided. However, studies of bat cell lines are worthy of mention here as they have demonstrated the mechanisms through which bat immune systems tolerate viral infections while maintaining bodily functions, which may provide useful insights into the management of persistent infection in severe COVID‐19. These features of bats include decreased induction of NLRP3 inflammasomes (Ahn et al., 2019) and constitutive ubiquitous expression of antiviral IFN‐ɑ (Zhou et al., 2016), implicating these mediators as of potential interest for the suppression of harmful inflammatory responses to viral infection or for inducing reductions in viral load.

It is also notable that SARS‐CoV‐2 isolates have shown infectivity in rabbit and pig cells in vitro (Chu et al., 2020). Although in vitro tropism does not always mean infection can occur in vivo, these model organisms may be useful for studies related to COVID‐19, as rabbits are well suited for longitudinal lung function studies and the organ systems of pigs more closely resemble the scale of those in humans which is useful for studies involving haemodynamic measurements.

## 4 APPROACHES TO IMPROVE ANIMAL MODELS OF PATHOLOGICAL MECHANISMS IN COVID‐19

### 4.1 Mode of inoculation with SARS‐CoV‐2

The route or method used for viral inoculation can affect the severity of viral disease models, and a range of inoculation routes have been used in reported animal studies (Table 1). Intranasal delivery has been widely used as this delivery method is simple, some inoculum can be aspirated into airways, and because the intranasal route is potentially reflective of the real‐world route of entry for viral droplets. However, this method can lead to limited and focal delivery of inoculum to lungs (Su, Looney, Robriquet, Fang, & Matthay, 2004), and viral diseases affecting the lower respiratory tract such as COVID‐19 may result from inhalation of aerosols which can enter deeper into the lungs or from widespread deposition of virus in lungs due to high upper respiratory tract viral load and mucociliary escalator disruption.

Aerosolised delivery of viral inoculum may therefore be useful in the induction of widespread lung injury which might be sufficient to provoke severe disease, as well as in the standardisation of the dose of inoculum that reaches the lower airways and respiratory lungs. Evidence that aerosolised delivery can provoke injury resembling severe ARDS with elevated cytokine release has been produced in cynomolgus macaque models of H5N1 influenza (Wonderlich et al., 2017). There have also been recent developments in the quantitative delivery of aerosols and powders to lungs of laboratory animals (Lexmond, Keir, Terakosolphan, Page, & Forbes, 2018). Aerosolised or intratracheal administration approaches for controlled delivery of inoculum may be worthy of consideration in animal models for more severe COVID‐19.

### 4.2 Demonstration of ARDS‐like lung pathology in animals

Histological analysis, superficial visual inspection, and radiological imaging have been the predominant approaches used to assess the development of lung inflammation and injury following experimental SARS‐CoV‐2 infection. However, these pathological observations have so far often been reported in only some animals under study and only some regions of the lungs of those animals (Table 1). This is likely to be due to probabilistic provocation of injury and primary focus of studies on early viral infection and transmission. For efficacy studies, it is important that disease‐relevant endpoints are measured in such a way that effects of potential therapeutics on features of ARDS can be statistically determined. Several reviews have established clear criteria for assessing ARDS‐relevant pathological features in laboratory animal ARDS models (Aeffner, Bolon, & Davis, 2015; Matute‐Bello et al., 2011). In order to demonstrate and measure extent of lung injury or ARDS, the ideal standard is to provide evidence of (a) visual lung injury and inflammation, (b) functional alteration of alveolar capillary barrier function, and (c) physiological dysfunction such as increased alveolar‐arterial oxygen tension difference (decreased PaO_2_/FiO_2_).

So far, repeated quantifications relevant to ARDS have not been demonstrated in animal models of SARS‐CoV‐2 infection, with the exception of one macaque study where pulmonary oedema was measured consistently in infected animals using the index of lung weight as a percentage of body weight (Munster et al., 2020). This simple method may prove to be a useful terminal approach for lung injury assessment that is feasible in most studies within the constraints of biosafety level (BSL) 3 conditions and later requirement for lung homogenate for viral load readouts.

Personnel time, the personal protective equipment requirements in BSL3 laboratories and both the size of animals and consideration of their welfare can limit measurements that are feasible in SARS‐CoV‐2 infection studies. However, serial measurements of blood oxygen saturation or arterial blood gases would be highly valuable in definition of the time course of impairment of gas exchange, if this occurs in animal models. It is notable here that, in COVID‐19 patients, decreased arterial oxygen saturation has been reported prior to the dyspnoea and dramatically decreased saturation which leads to hospitalisation and PaO_2_/FiO_2_ can fluctuate during the course of clinical COVID‐19 progression, and so, as in the clinical setting, serial close monitoring of oxygen saturation is ideal (Cascella, Rajnik, Cuomo, Dulebohn, & Di Napoli, 2020). These points are reminders that some degree of hypoxaemia and pneumonia is indicative of some disease but not conclusive evidence of progression to severe COVID‐19‐like ARDS.

Where possible, serial non‐invasive imaging approaches as reported in some macaque studies (Table 1) are also useful for tracking the time course of pulmonary oedema development. It is notable that some patients with COVID‐19 ARDS have more limited oedema with severe hypoxaemia (Gattinoni et al., 2020), and so additional measurements of lung perfusion (pulmonary dead space fraction or ventilation perfusion scans) in animals may also be useful for better understanding of COVID‐19 as disease sub‐phenotypes may be also be present within animal models (Carla et al., 2020).

Planning of terminal measurements should involve consideration of how the maximum number of high‐value measurements can be made. If inoculations can affect the lungs widely and relatively evenly, this can involve the use of different lobes for different potentially confounding measurements. For example, bronchoalveolar lavage is useful for simultaneous measurements of viruses, inflammatory cells, inflammatory mediators, and protein deposits in the bronchoalveolar spaces but causes artefacts in histological analysis and prevents later measurement of pulmonary oedema. Likewise, formalin inflation is useful for histological analysis which can be performed outside BSL3 conditions but prevents the most direct and quantitative methods of measurement of viral load in lungs and pulmonary oedema. Lobes of lungs can therefore be sequentially tied off and sampled to permit functional and histological measurements related to ARDS as well as viral load in lungs. Experimental designs will of course require appropriate trade‐offs between study complexity and welfare and safety of animals and laboratory workers.

### 4.3 Non‐BSL3 approaches for modelling mechanisms relevant to COVID‐19

The serious and potentially lethal nature of COVID‐19 means that BSL3 laboratories are required for experiments using SARS‐CoV‐2. However, insights into mechanisms driving COVID‐19 may still be derived from non‐infectious models.

One example directly related to SARS‐CoV‐2 is the effects of absence of ACE2 function on enhancement of the pulmonary oedema response, which were established using a mouse acid inhalation model which to provoke rapid and severe pulmonary oedema (Kuba et al., 2005). This finding has led to ongoing trials to assess whether administration of recombinant ACE2 may address infection‐related deficiencies in lung barrier protective ACE2‐derived Ang 1‐7 generation from angiotensin II in addition to its potential action as a decoy preventing viral entry into cells (Sriram & Insel, 2020).

Some urgently needed studies of putative therapeutic agents for COVID‐19, particularly those targeting host responses, may therefore be possible or supportive using other lung injury models with higher throughput. Influenza viruses have been adapted to infect mouse cells and can be used for viral induced, lethal lung injury experiments in mice in more widely available BSL2 laboratories where the mechanisms under study might feasibly be shared in SARS‐CoV‐2 responses. However, immune responses to coronaviruses and influenza are not the same, as for example knockout of complement component 3 in mice is detrimental in influenza H1N1 and H5N1 infection models (O'Brien, Morrison, Dundore, Heise, & Schultz‐Cherry, 2011) but beneficial in SARS‐CoV infection models (Gralinski et al., 2018). Viral pseudotypes expressing SARS‐CoV‐2 Spike may also be useful for in vivo non‐BSL3 studies of antiviral efficacy (Hoffmann et al., 2020), although these will not model the evasion mechanisms and immunopathology unique to SARS‐CoV‐2 or related coronaviruses.

There are many other models used for the study of ARDS in mice and other model organisms closer phylogenetically and anatomically to humans (Aeffner et al., 2015; Matute‐Bello et al., 2011; Matute‐Bello, Frevert, & Martin, 2008). Of these, lung injury caused by instillations of bacteria or endotoxins, ventilation, and antibodies may be particularly relevant to COVID‐19 complicated by secondary infections, barotrauma and antibody reactions, and associated dysregulated responses. Additionally, there may be central mechanisms of ARDS progression and resolution meaning that non‐viral models may have utility for rapid proof‐of‐concept studies outside BSL3 conditions, although it is likely that in many cases efficacy studies with SARS‐CoV‐2 infection will also be needed.

### 4.4 Incorporating known COVID‐19 risk factors into models

Known risk factors for severe COVID‐19 offer opportunities to drive models towards pathological states (Figure 1). Advanced age, obesity, and diabetes are additional risk factors associated with high case‐fatality rates COVID‐19 (Onder, Rezza, & Brusaferro, 2020; Petrilli et al., 2020). These features may predispose towards severe disease through complicating medical treatment, by hypoventilation as a result of low cardiorespiratory fitness, and through dysregulated immunity with imbalance of inflammation and repair mechanisms, as well as a propensity for underlying cardiovascular disease and changes to haemostasis that will influence progression of respiratory infection towards ARDS (Sattar, McInnes, & McMurray, 2020; Tzoran, Hoffman, & Monreal, 2018).

The use of aged animals might allow for modelling of the age risk factor. It is encouraging in this light that early development of preclinical models of COVID‐19 includes the influence of age in rhesus macaques. Infection with SARS‐CoV‐2 reportedly led to heightened lung pathology and viral replication in older macaques (15 years old) compared to younger animals (5 years old) (Yu et al., 2020). Additionally, aged mice have previously demonstrated exacerbated inflammation and lung injury following SARS‐CoV infection (Rockx et al., 2009).

Animal models which require ageing over years may not permit the rapid testing of treatments which is required for the COVID‐19 pandemic. Models incorporating other risk factors such as induced obesity (e.g., high fat diet‐induced), diabetes (e.g., leptin receptor deficiency; Paul, Queen, Page, & Ferro, 2007), or impaired immune responses (e.g., STAT1 knockout/inhibition; Frieman et al., 2010) might be made available more quickly. Additionally, COVID‐19 may progress to severe disease status as a result of immune priming, secondary infections, or intravascular sequelae, and so combining SARS‐CoV‐2 infection models with a first or second hit such as immune priming with LPS or additional challenges such as the non‐BSL3 approaches to cause lung injury described above (i.e., inoculation with pathogenic bacteria or injections of injurious antibodies) might be capable of driving host responses towards a pathological state.

### 4.5 Platelet responses, coagulopathy, and hyperinflammation

As discussed above, severe COVID‐19 is also associated with low blood platelet counts and thrombosis, a consumptive coagulopathy, and a hyperinflammatory state involving platelet activation, release of NETs, and prolonged systemic elevations of cytokines such as IL‐6 and CXCL10. These pathological features associated with severe disease have not yet been demonstrated in animal models (Table 1).

The degree of thrombocytopaenia has been reported as a potential biomarker for severe COVID‐19 (Lippi, Plebani, & Henry, 2020). A large retrospective study revealed the degree of thrombocytopaenia to be dynamic after presentation to clinic, with decreasing platelet numbers being predictive of mortality later on (Liu et al., 2020). Furthermore, a shift in platelet‐lymphocyte ratio may indicate the occurrence of an acute inflammatory or thrombotic event and therefore have prognostic value (Qu et al., 2020). Thus, thrombocytopaenia may reflect (a) alteration in thrombopoiesis due to the bone marrow or lungs (and potentially spleen) being inflamed or receiving inflammatory and trauma‐related thrombopoietic cues; (b) localised lung recruitment of platelets as a facet of their role in the immune response or alveolar coagulation; (c) disseminated intravascular coagulation (DIC) throughout the body (Xu, Zhou, & Xu, 2020); or (d) platelet‐viral interaction, although this remains hypothetical as an engagement of platelets with SARS‐CoV‐2 has not been described (Amgalan & Othman, 2020). Evidence from other disease states with features of COVID‐19 pathology indicate potential causative roles of platelets in the worsening of the disease, which might therefore provide opportunities for adoption of more severe mechanistic models of severe COVID‐19. Reports on animal studies published to date have not included measurements of these pathological responses, but their detection would be useful in further evidence for successful modelling of mechanisms of severe disease.

Results of studies in mice modelling influenza agree on the necessity for platelets in the immune response and inflammation but conflict as to whether this is beneficial (Campbell et al., 2019; Guo et al., 2017), or detrimental (Boilard et al., 2014; Lê et al., 2015). Additionally, platelets respond to influenza virus by increasing complement availability and encourage the release of NETs into blood, and so platelets may be important integrators linking viral infection to neutrophil responses that are associated with coagulopathy and venous thrombosis (Koupenova et al., 2019). Furthermore, platelets have a complex relationship with lung inflammation in that they can be both protective of the alveolar capillary barrier or can promote excessive vascular leak (Middleton, Rondina, Schwertz, & Zimmerman, 2018; Weyrich & Zimmerman, 2013).

Given the importance of platelets in coagulopathies (DIC, and alveolar thrombi), the innate immune response and hyperinflammation, their influence on the patency of the alveolar capillary unit, and the association of low blood platelet counts with severe disease; the manipulation of platelets might be a useful experimental tool to replicate these pathological events in animal models of SARs‐CoV‐2 infection. Our previous findings that experimental thrombocytopenia can convert a mild self‐resolving bacterial lung infection to a more severe form of systemic infection, with extra‐pulmonary organ involvement and death (Amison et al., 2018) suggest that the induction of severe experimental thrombocytopenia (>95% depletion) in animal models of SARS‐CoV‐2 may allow these infection models to demonstrate the progression from a mild to a severe disease phenotype. Additionally, pulmonary platelet retention can be induced by intravenous injections with anti‐MHC class I antibodies which are also associated with thrombocytopenia and release of NETs (Caudrillier et al., 2012; Looney et al., 2009). The use of these antibodies combined with infection models might be useful in modelling the contributions of platelets and NETs to severe COVID‐19 associated with lung hypoperfusion, release of NETs, and autoantibody production (Zhang, Xiao, et al., 2020). The involvement of FcγRIIA in viral platelet responses suggests that transgenic expression of human FcγRIIA on platelets may be useful in driving greater platelet responses in murine SARS‐CoV‐2 infection models, as mice lack activating Fcγ receptors on platelets (Boilard et al., 2014).

Treatment with heparin or low MW heparin has been associated with reduced mortality in clinical studies of COVID‐19 (Tang et al., 2020; Yin, Huang, Li, & Tang, 2020), and nebulised delivery of heparin is associated with a reduced requirement for ventilation in patients hospitalised by other respiratory diseases such as severe COPD (Ashoor, Hasseb, & Esmat, 2020; Dixon, Schultz, Hofstra, Campbell, & Santamaria, 2010; Shute, Puxeddu, & Calzetta, 2018). Although heparin is classically used as an anticoagulant, it has also been demonstrated to bind and reduce the activity of a range of cytokines implicated in the COVID‐19‐associated cytokine storm (Mulloy, Hogwood, Gray, Lever, & Page, 2015) and also interacts with the SARS‐CoV‐2 Spike protein in a manner which causes structural alteration of the ACE2‐binding domain which is likely to reduce viral entry (Mycroft‐West et al., 2020). The potentially useful polypharmacology of heparin underlines the need for integrated in vivo SARS‐CoV‐2 infection models incorporating simultaneous and clinically relevant measurements of coagulation, cytokines and viral load.

In addition to platelet number, the severity of COVID‐19 disease progression also correlates closely with levels of the pro‐inflammatory mediators such as CXCL10 (IFN‐inducible protein 10) and TNFα, both associated with the pathology and progression associated with hyperinflammatory condition associated with ARDS (Bautista et al., 2013; Tang et al., 2020; Yang et al., 2020). The amelioration of LPS induced lung inflammation following CXCL10 neutralisation in rats, and the proposition of anti‐TNFα therapy in COVID‐19 patients, therefore highlights the potential for combining SARS‐CoV‐2 infections with TNFα and CXCL10 challenge as a further method in the development of models with a more severe phenotype. (Feldmann et al., 2020; Lang et al., 2017).

Persistence of infection and inflammation due to ineffective viral clearance is a key feature of severe COVID‐19, although maintenance of infection and systemic cytokine release has not yet been reported in animal models of SARS‐CoV‐2 infection. A potential approach to model this feature of severe disease is the experimental disruption of important antiviral host defence pathways. A key mediator of defensive signalling might be the transcription factor STAT1, the genetic deletion of which can convert a mild SARS‐CoV mouse infection model into one resulting in 100% mortality (Frieman et al., 2010). Pharmacological inhibitors of STAT1 are available (Miklossy, Hilliard, & Turkson, 2013) and may be useful in the rapid development of animal models with deficiencies in protective antiviral responses, without the requirement for multigenerational crosses or ageing of laboratory animals.

Dysregulated immune responses by myeloid leukocytes may also be important in pathogenesis of severe disease (Barnes et al., 2020). Although the impact of COVID‐19 on cystic fibrosis patients is still unclear (Colombo et al., 2020), mouse models of cystic fibrosis mutations involve heightened platelet activation and a hyperinflammatory state with increased neutrophil responses, so these models might be useful in driving increased severity of pathology (Ortiz‐Muñoz et al., 2020).

### 4.6 Time course of interventions relative to disease progression

Efficacy studies using animal models of many disease processes have been criticised for over‐use of the prophylactic application of interventions (i.e., pretreatment) when such approaches would not be realistic for clinical treatments (Denayer, Stöhrn, & Van Roy, 2014). Severe COVID‐19 has a characteristic time course which suggests that there are several windows where different treatment approaches might be useful (Zhou et al., 2020) (Figure 2).

The users of animal models for COVID‐19 research should wherever possible assess the time course of readouts in their models and consider the type of therapy being assessed, the potential of risk to patients from any anticipated adverse effects, and whether the intervention is likely to need to take place in a hospital setting. For example, as viral replication increases, likelihood of transmission and persistent infection mediates severe disease, thus antivirals with low toxicity might be useful for all diagnosed cases and perhaps even prophylactically in high‐risk populations. Some interventions such as nebulised heparin may be of benefit as antiviral agents (Mycroft‐West et al., 2020) and in reducing the inflammatory response (Dixon et al., 2010; Mulloy et al., 2015), and they may need to be dosed at different time points across the development of COVID‐19. Once severe disease develops, interventions which are aimed at resolving ARDS and systemic complications would be useful, and robust preclinical testing might require models which include aspects of intensive care support such as invasive ventilation (Alexander et al., 2020). Careful model characterisation will be required to identify analogous phases of pathology development in animal models for appropriate timing of interventions under study.

**TABLE 3:** Limitations of animal models of COVID‐19 and potential opportunities for model development

Limitation of animal model approach	Opportunity for model development
• Limited availability and bandwidth of BSL3 laboratories	• Studies using viral pseudotypes • Do some tests in non‐BSL3 models reproducing related mechanisms
• No infectivity of SARS‐CoV‐2 in model species	• Humanised ACE2 transgenics • Adaptation of virus
• Limited or patchy lung pathology and lack of viral persistence and systemic sequelae	• Use of immunodeficiency models • Studies using aged animals • Aerosolised delivery of viral inoculum potentially at higher titres • Use additional triggers of lung injury to distribute pathology more widely
• Model poorly predictive of success of intervention in clinic	• Apply intervention at clinically feasible time • Measure disease‐relevant endpoints • Use model organism closer in phylogeny to humans

## 5 CONCLUSION

Animal models have been rapidly mobilised to address the need for greater understanding of COVID‐19 and for testing of new therapeutic approaches to this pandemic. While many useful observations have already been shared, it is important that animal studies are adapted to measure and report key readouts that are relevant to clinical COVID‐19 such as evidence of lung injury resembling ARDS, physiological gas exchange impairment, hyperinflammation, platelet responses, and coagulopathy. Study designs may need to incorporate additional risk factors or disease triggers to shift from modelling protective and self‐resolving infection and inflammation to replicating severe disease with ARDS and multi‐organ failure (Table 3). Development of models that reproduce these hallmarks of disease will permit better understanding of COVID‐19 pathogenesis and facilitate improved efficacy testing of desperately needed drugs and other therapeutic actions.

### Nomenclature of targets and ligands

Key protein targets and ligands in this article are hyperlinked to corresponding entries in http://www.guidetopharmacology.org, the common portal for data from the IUPHAR/BPS Guide to PHARMACOLOGY (Harding et al., 2018), and are permanently archived in the Concise Guide to PHARMACOLOGY 2019/20 (Alexander et al., 2019).

## CONFLICT OF INTEREST

The authors declare no conflicts of interest.
